# Variants associated with *HHIP* expression have sex-differential effects on lung function

**DOI:** 10.12688/wellcomeopenres.15846.2

**Published:** 2021-05-24

**Authors:** Katherine A. Fawcett, Ma'en Obeidat, Carl Melbourne, Nick Shrine, Anna L. Guyatt, Catherine John, Jian'an Luan, Anne Richmond, Marta R. Moksnes, Raquel Granell, Stefan Weiss, Medea Imboden, Sebastian May-Wilson, Pirro Hysi, Thibaud S. Boutin, Laura Portas, Claudia Flexeder, Sarah E. Harris, Carol A. Wang, Leo-Pekka Lyytikäinen, Teemu Palviainen, Rachel E. Foong, Dirk Keidel, Cosetta Minelli, Claudia Langenberg, Yohan Bossé, Maarten Van den Berge, Don D. Sin, Ke Hao, Archie Campbell, David Porteous, Sandosh Padmanabhan, Blair H. Smith, David M. Evans, Sue Ring, Arnulf Langhammer, Kristian Hveem, Cristen Willer, Ralf Ewert, Beate Stubbe, Nicola Pirastu, Lucija Klaric, Peter K. Joshi, Karina Patasova, Mangino Massimo, Ozren Polasek, John M. Starr, Stefan Karrasch, Konstantin Strauch, Thomas Meitinger, Igor Rudan, Taina Rantanen, Kirsi Pietiläinen, Mika Kähönen, Olli T. Raitakari, Graham L. Hall, Peter D. Sly, Craig E. Pennell, Jaakko Kaprio, Terho Lehtimäki, Veronique Vitart, Ian J. Deary, Debbie Jarvis, James F. Wilson, Tim Spector, Nicole Probst-Hensch, Nicholas J. Wareham, Henry Völzke, John Henderson, David P. Strachan, Ben M. Brumpton, Caroline Hayward, Ian P. Hall, Martin D. Tobin, Louise V. Wain

**Affiliations:** 1 Department of Health Sciences, University of Leicester, Leicester, LE1 7RH, UK; 2 The University of British Columbia Centre for Heart Lung Innovation, St Paul’s Hospital, Vancouver, BC, Canada; 3 MRC Epidemiology Unit, University of Cambridge School of Clinical Medicine, Cambridge, CB2 0QQ, UK; 4 MRC Human Genetics Unit, Institute of Genetics and Molecular Medicine, University of Edinburgh, Western General Hospital, Crewe Road, Edinburgh, EH4 2XU, UK; 5 K.G. Jebsen Center for Genetic Epidemiology, Department of Public Health and Nursing, NTNU, Norwegian University of Science and Technology, Trondheim, Norway; 6 Medical Research Council Integrative Epidemiology Unit, University of Bristol, Bristol, BS8 2BN, UK; 7 Department of Functional Genomics, Interfaculty Institute for Genetics and Functional Genomics, University Medicine Greifswald, Greifswald, 17475, Germany; 8 Swiss Tropical and Public Health Institute, Basel, Switzerland; 9 University of Basel, Basel, Switzerland; 10 Centre for Global Health Research, Usher Institute, University of Edinburgh, Teviot Place, Edinburgh, EH8 9AG, UK; 11 The Department of Twin Research & Genetic Epidemiology, King’s College London, St Thomas’ Campus, Lambeth Palace Road, London, UK; 12 Population Health and Occupational Disease, National Heart and Lung Institute, Imperial College London, London, UK; 13 Institute of Epidemiology, Helmholtz Zentrum München - German Research Center for Environmental Health, Neuherberg, 85764, Germany; 14 Centre for Cognitive Ageing and Cognitive Epidemiology, University of Edinburgh, Edinburgh, EH8 9JZ, UK; 15 Psychology, University of Edinburgh, Edinburgh, EH8 9JZ, UK; 16 School of Medicine and Public Health, Faculty of Medicine and Health, The University of Newcastle, Callaghan, Australia; 17 Department of Clinical Chemistry, Fimlab Laboratories, Tampere, 33520, Finland; 18 Department of Clinical Chemistry, Finnish Cardiovascular Research Center - Tampere, Faculty of Medicine and Health Technology, Tampere University, Tampere, 33014, Finland; 19 Department of Cardiology, Heart Center, Tampere University Hospital, Tampere, 33521, Finland; 20 Institute for Molecular Medicine FIMM, University of Helsinki, Helsinki, FI-00014, Finland; 21 Telethon Kids Institute, Perth, Australia; 22 School of Physiotherapy and Exercise Science, Faculty of Health Sciences, Curtin University, Perth, Australia; 23 Institut universitaire de cardiologie et de pneumologie de Québec, Department of Molecular Medicine, Laval University, Québec, Canada; 24 University Medical Center Groningen, Department of Pulmonology, GRIAC Research Institute, University of Groningen, Groningen, The Netherlands; 25 Respiratory Division, Department of Medicine, University of British Columbia, Vancouver, BC, Canada; 26 Department of Genetics and Genomic Sciences, Icahn School of Medicine at Mount Sinai, New York, USA; 27 Centre for Genomic and Experimental Medicine, Institute of Genetics and Molecular Medicine, Western General Hospital, Edinburgh, EH4 2XU, UK; 28 British Heart Foundation Glasgow Cardiovascular Research Centre, Institute of Cardiovascular and Medical Sciences, College of Medical, Veterinary and Life Sciences, University of Glasgow, Glasgow, G12 8TA, UK; 29 Division of Population Health Sciences, Ninewells Hospital and Medical School, University of Dundee, Dundee, DD1 9SY, UK; 30 Population Health Sciences Bristol Medical School, University of Bristol, Bristol, BS8 2BN, UK; 31 University of Queensland Diamantina Institute, Translational Research Institute, Brisbane, QLD 4072, Australia; 32 Department of Public Health and Nursing, Faculty of Medicine and Health Sciences, NTNU, Norwegian University of Science and Technology, Trondheim, Norway; 33 Department of Biostatistics and Center for Statistical Genetics, University of Michigan, Ann Arbor, USA; 34 Department of Internal Medicine, University of Michigan, Ann Arbor, USA; 35 Department of Human Genetics, University of Michigan, Ann Arbor, USA; 36 Department of Internal Medicine B, Cardiology, Pneumology, Infectious Diseases, Intensive Care Medicine, University Medicine Greifswald, Greifswald, 17475, Germany; 37 University of Split School of Medicine, Split, Croatia; 38 Alzheimer Scotland Research Centre, University of Edinburgh, Edinburgh, EH8 9JZ, UK; 39 Institute and Outpatient Clinic for Occupational, Social and Environmental Medicine, Ludwig-Maximilians-Universität, Munich, 80336, Germany; 40 Institute of Epidemiology, Helmholtz Zentrum München - German Research Center for Environmental Health, Neuherberg, 85764, Germany; 41 Comprehensive Pneumology Center Munich (CPC-M), Member of the German Center for Lung Research (DZL), Munich, 81377, Germany; 42 Institute of Genetic Epidemiology, Helmholtz Zentrum München, German Research Center for Environmental Health, Neuherberg, 85764, Germany; 43 Chair of Genetic Epidemiology, IBE, Faculty of Medicine, LMU Munich, Munich, 81377, Germany; 44 Institute of Human Genetics, Helmholtz Zentrum München, German Research Center for Environmental Health, Neuherberg, 85764, Germany; 45 Institute of Human Genetics, Klinikum rechts der Isar der TU Muenchen, Muenchen, 81675, Germany; 46 Faculty of Sport and Health Sciences, Gerontology Research Center, University of Jyväskylä, Jyväskylä, Finland; 47 Obesity Research Unit, Research Program for Clinical and Molecular Metabolism, Faculty of Medicine, University of Helsinki, Helsinki, FI-00014, Finland; 48 Obesity Centre, Abdominal Centre, Helsinki University Hospital and University of Helsinki, Helsinki, FI-00029, Finland; 49 Department of Clinical Physiology, Tampere University Hospital, Tampere, 33521, Finland; 50 Department of Clinical Physiology, Finnish Cardiovascular Research Center - Tampere, Faculty of Medicine and Health Technology, Tampere University, Tampere, 33014, Finland; 51 Centre for Population Health Research, University of Turku and Turku University Hospital, Turku, Finland; 52 Research Centre of Applied and Preventive Cardiovascular Medicine, University of Turku, Turku, Finland; 53 Department of Clinical Physiology and Nuclear Medicine, Turku University Hospital, Turku, Finland; 54 Children's Health and Environment Program, The University of Queensland, Brisbane, Australia; 55 Department of Public Health, University of Helsinki, Helsinki, FI-00014, Finland; 56 MRC-PHE Centre for the Environment and Health, London, UK; 57 Intitute for Community Medicine, University Medicine Greifswald, Greifswald, 17487, Germany; 58 Population Health Research Institute, St George's, University of London, London, SW17 0RE, UK; 59 Clinic of Thoracic and Occupational Medicine, St. Olavs Hospital, Trondheim University Hospital, Trondheim, Norway; 60 MRC Integrative Epidemiology Unit, University of Bristol, Bristol, UK; 61 Division of Respiratory Medicine and NIHR-Nottingham Biomedical Research Centre, University of Nottingham, Nottingham, UK; 62 National Institute for Health Research, Leicester Respiratory Biomedical Research Centre, Glenfield Hospital, Leicester, LE3 9QP, UK

**Keywords:** genome-wide interaction study, lung function, sex, HHIP, expression

## Abstract

**Background: **Lung function is highly heritable and differs between the sexes throughout life. However, little is known about sex-differential genetic effects on lung function. We aimed to conduct the first genome-wide genotype-by-sex interaction study on lung function to identify genetic effects that differ between males and females.

**Methods:** We tested for interactions between 7,745,864 variants and sex on spirometry-based measures of lung function in UK Biobank (N=303,612), and sought replication in 75,696 independent individuals from the SpiroMeta consortium.

**Results:** Five independent single-nucleotide polymorphisms (SNPs) showed genome-wide significant (P<5x10 ^-8^) interactions with sex on lung function, and 21 showed suggestive interactions (P<1x10 ^-6^). The strongest signal, from rs7697189 (chr4:145436894) on forced expiratory volume in 1 second (FEV _1_) (P=3.15x10 ^-15^), was replicated (P=0.016) in SpiroMeta. The C allele increased FEV _1_ more in males (untransformed FEV _1_ β=0.028 [SE 0.0022] litres) than females (β=0.009 [SE 0.0014] litres), and this effect was not accounted for by differential effects on height, smoking or pubertal age. rs7697189 resides upstream of the hedgehog-interacting protein ( *HHIP*) gene and was previously associated with lung function and *HHIP* lung expression. We found *HHIP* expression was significantly different between the sexes (P=6.90x10 ^-6^), but we could not detect sex differential effects of rs7697189 on expression.

**Conclusions:** We identified a novel genotype-by-sex interaction at a putative enhancer region upstream of the *HHIP* gene. Establishing the mechanism by which *HHIP* SNPs have different effects on lung function in males and females will be important for our understanding of lung health and diseases in both sexes.

## Introduction

Measures of lung function, including forced expiratory volume in 1 second (FEV _1_) and forced vital capacity (FVC), are used to determine diagnosis and severity of chronic obstructive pulmonary disease (COPD). COPD refers to a group of complex lung disorders characterised by irreversible (and usually progressive) airway obstruction, and is projected to be the third leading cause of death globally in 2020 ^1^. The major risk factor for COPD is smoking, but other environmental and genetic factors have been identified.

Physiological lung development and function differ throughout life between males and females ^2^. It is known that sex hormones can influence these processes but the mechanisms are not well understood ^3, 4^. The incidence and presentation of lung diseases such as COPD also exhibit sexual dimorphism. Traditionally viewed as a disease of older males, COPD has been increasing in prevalence amongst females over the last two decades. It has been reported that females are more vulnerable to environmental risk factors for COPD and are over-represented amongst sufferers of early-onset severe COPD ^5, 6^. Females are also more likely to present with small airway disease whereas males are more likely to develop emphysematous phenotype. Moreover, females report more frequent and/or severe exacerbations of respiratory symptoms than males and higher levels of dyspnoea and cough ^5^.

In a recent paper, 279 genetic loci were reported as associated with lung function traits, but these only explain a small proportion of the heritability ^7^. One possible source of hidden heritability is the interaction between genetic factors and biological sex on lung function traits. A genome-wide genotype-by-sex interaction study in three studies comprising 6260 COPD cases and 5269 smoking controls found a putative sex-specific risk factor for COPD in the *CELSR1* gene, a region not previously implicated in COPD or lung function ^8^. However, having sufficient statistical power to reproducibly detect genotype-by-sex interactions requires much larger sample sizes. Statistical power can also be enhanced by using quantitative lung function traits as outcomes instead of COPD diagnoses, but we are not aware of any genome-wide genotype-by-sex interaction studies on lung function traits. Understanding the role of sex in lung function and COPD will be important for developing therapeutics that work for both males and females ^9^.

In this study, we tested for an interaction effect of 7,745,864 variants and sex on FEV _1_, FEV _1_/FVC, FVC and PEF in 303,612 individuals from the UK Biobank resource. We sought replication of our findings in 75,696 independent individuals from the SpiroMeta consortium. To our knowledge this is the first genome-wide sex-by-genotype interaction study on lung function traits, and the largest sex-by-genotype interaction study to focus on COPD-related outcomes.

## Results

We tested 7,745,864 genome-wide variants with minor allele frequency (MAF) ≥ 0.01 and imputation quality scores ≥ 0.3 for genotype-by-sex interactions on lung function in 303,612 unrelated individuals of European ancestry from UK Biobank. Five independent signals were identified showing genome-wide significant (P<5 x 10 ^-8^) interaction with sex on at least one of four lung function traits (FEV _1_, FEV _1_/FVC, FVC, and PEF) with a further 21 SNPs showing suggestive significance (P<1 x 10 ^-6^) ( Table 1; Figure S1, *Extended data* ^10^). The top three genome-wide significant signals had been previously reported for association with lung function: rs7697189 near the gene encoding hedgehog-interacting protein ( *HHIP*) (interaction P = 3.15 x 10 ^-15^), rs9403386 near the gene encoding Adhesion G Protein-Coupled Receptor G6 ( *ADGRG6*, previously known as *GPR126*) (interaction P = 4.56 x 10 ^-9^), and rs162185 downstream of the gene encoding transcription factor 21 ( *TCF21*) (interaction P = 4.87 x 10 ^-9^) ^11– 16^. This may, in part, reflect greater power to detect interactions with variants with strong main effects on lung function. Only rs355079 (interaction P = 8.84 x 10 ^-7^) showed significant effects in opposite directions in males compared to females.

**Table 1.** Association between top SNPs and lung function in males and females, and genotype-by-sex interaction results.

SNP (nearest gene) and coordinates	Test/ other allele	Trait	Lung function UK Biobank males			Lung function UK Biobank females			Sex interaction in UK Biobank		Sex interaction in SpiroMeta	
			MAF	Beta (SE)	P	MAF	Beta (SE)	P	Beta (SE)	P	Beta (SE)	P
rs7697189 (HHIP) 4:145436894	C/G	FEV _1_	0.390	0.052 (0.004)	2.13E-33	0.392	0.013 (0.003)	1.16E-05	-0.040 (0.005)	3.15E-15	**-0.025 (0.01)**	**0.016**
rs9403386 (ADGRG6) 6:142764073	C/A	FEV _1_/FVC	0.031	0.214 (0.012)	4.48E-75	0.031	0.128 (0.009)	2.16E-43	-0.086 (0.015)	4.56E-09	**-0.035 (0.032)**	**0.281**
rs162185 (TCF21) 6:134226147	C/T	PEF	0.411	-0.038 (0.004)	1.35E-18	0.410	-0.009 (0.003)	0.002	0.030 (0.005)	4.87E-09	**0.022 (0.0139)**	**0.083**
rs6480592 (CHST3) 10:73764509	C/T	PEF	0.398	-0.021 (0.004)	1.66E-06	0.400	0.007 (0.003)	0.011	0.028 (0.005)	2.85E-08	**0.003 (0.012)**	**0.808**
rs111893604 (ZSCAN10) 16:3141104	G/T	FEV _1_	0.059	0.040 (0.009)	1.70E-05	0.059	-0.020 (0.006)	0.002	-0.060 (0.011)	4.04E-08	0.006 (0.026)	0.827
rs72694266 (RP11-907D1.1) 14:97578576	A/C	PEF	0.077	-0.044 (0.008)	2.69E-07	0.078	0.008 (0.006)	0.145	0.053 (0.010)	6.31E-08	-0.049 (0.027)	0.066
rs72781459 10:10247676	C/T	PEF	0.096	0.031 (0.007)	3.44E-05	0.097	-0.012 (0.005)	0.014	-0.046 (0.009)	1.08E-07	0.007 (0.021)	0.729
rs74316059 (RP11-649A16.1) 3:146983325	T/C	FEV _1_/FVC	0.042	0.049 (0.010)	2.52E-06	0.043	-0.018 (0.008)	0.029	-0.068 (0.013)	2.38E-07	**-0.031 (0.028)**	**0.269**
rs55789572 (EIF2S2/RALY) 20:32687822	A/C	FEV _1_	0.022	0.041 (0.015)	0.006	0.022	-0.047 (0.010)	2.67E-06	-0.089 (0.017)	2.80E-07	**-0.01 (0.033)**	**0.765**
rs74933518 (DAPK2) 15:64303295	A/G	PEF	0.025	-0.072 (0.014)	1.23E-07	0.025	0.007 (0.009)	0.421	0.082 (0.016)	3.05E-07	**0.025 (0.043)**	**0.568**
rs11247571 (ABR) 17:908502	G/A	PEF	0.343	-0.025 (0.005)	3.65E-08	0.344	0.002 (0.003)	0.569	0.027 (0.005)	3.22E-07	**0.010 (0.014)**	**0.473**
rs707588 (RP11-154H17.1) 1:5711430	G/A	FEV _1_	0.482	-0.020 (0.004)	3.23E-06	0.482	0.006 (0.003)	0.029	0.025 (0.005)	3.27E-07	**0.014 (0.01)**	**0.183**
rs138473298 (AUTS2) 7:69644989	T/C	PEF	0.012	-0.077 (0.020)	0.0002	0.011	0.043 (0.014)	0.002	0.122 (0.024)	3.52E-07	**0.037 (0.060)**	**0.540**
rs139069254 (RP11-648K4.2) 15:88113916	A/G	FEV _1_	0.018	0.071 (0.016)	1.83E-05	0.018	-0.027 (0.011)	0.017	-0.098 (0.019)	4.66E-07	**-0.051 (0.041)**	**0.216**
rs138163836 (PVRL3) 3:110952902	C/T	FVC	0.021	0.064 (0.015)	1.94E-05	0.020	-0.025 (0.011)	0.019	-0.091 (0.018)	5.07E-07	**-0.025 (0.038)**	**0.5**
rs28493055 (XDH) 2:31573390	T/G	FEV _1_	0.012	0.065 (0.020)	0.002	0.013	-0.055 (0.014)	6.40E-05	-0.119 (0.024)	5.60E-07	0.035 (0.054)	0.519
rs117380804 18:76145905	T/C	FVC	0.035	0.035 (0.012)	0.003	0.036	-0.035 (0.008)	1.93E-05	-0.070 (0.014)	6.25E-07	**-0.034 (0.03)**	**0.255**
rs602622 (RASGRP3) 2:33658226	C/G	PEF	0.444	-0.022 (0.004)	2.11E-07	0.445	0.002 (0.003)	0.444	0.025 (0.005)	6.45E-07	-0.013 (0.013)	0.323
rs2253718 (RF00019, SFTA2) 6:30900427	T/G	PEF	0.409	-0.049 (0.004)	5.69E-30	0.405	-0.027 (0.003)	1.78E-20	0.025 (0.005)	7.05E-07	**0.002 (0.016)**	**0.925**
rs2353939 (HHIP) 4:145729724	G/A	FVC	0.437	0.016 (0.004)	0.0002	0.435	-0.009 (0.003)	0.002	-0.025 (0.005)	7.55E-07	**-0.016 (0.01)**	**0.124**
rs7691139 (ZNF280A) 22:22876151	G/C	FEV _1_/FVC	0.116	-0.025 (0.007)	0.0003	0.115	0.017 (0.005)	0.002	0.043 (0.009)	7.62E-07	Not tested	
rs13020954 2:17296984	C/T	FEV _1_/FVC	0.014	0.050 (0.017)	0.004	0.014	-0.057 (0.014)	3.83E-05	-0.109 (0.022)	7.88E-07	**-0.062 (0.043)**	**0.148**
rs2731120 (MLF1) 3:158297633	A/C	FVC	0.346	0.029 (0.004)	3.72E-11	0.346	0.003 (0.003)	0.310	-0.026 (0.005)	8.14E-07	**-0.008 (0.011)**	**0.433**
rs355079 (LMCD1-AS1) 3:8643371	T/C	FVC	0.337	0.015 (0.004)	0.0007	0.339	-0.011 (0.003)	0.0004	-0.026 (0.005)	8.84E-07	0.001 (0.011)	0.935
rs7338055 (SPRYD7) 13:50504226	C/A	FVC	0.259	0.018 (0.005)	0.0001	0.259	-0.009 (0.003)	0.008	-0.028 (0.006)	9.81E-07	**-0.008 (0.012)**	**0.478**
rs34490170 (NEUROD1/ CERKL) 2:182576419	C/T	FVC	0.110	-0.035 (0.007)	6.41E-07	0.110	0.007 (0.005)	0.186	0.041 (0.008)	9.95E-07	**0.009 (0.018)**	**0.622**

The SNPs are those that demonstrate a sex-interaction effect on lung function in UK Biobank (P<1x10 ^-6^) (N = 303,612). Lung function traits were pre-adjusted for age, age ^2^, standing height and smoking status and the residuals rank-transformed to normality. The regression models also included genotyping array and the first ten ancestry-based principal components. For each SNP, columns 4-9 provide minor allele frequency (MAF), and beta-coefficients, standard errors and the P value for their association with lung function in males and females separately. Columns 10-11 show the results of the SNP-by-sex interaction in UK Biobank, where the effect is given in females relative to males. For example, the top SNP (rs7697189) shows a less positive effect in females compared to males and its beta coefficient is therefore negative. Columns 12-13 show the results of the SNP-by-sex interaction in 20 cohorts of the SpiroMeta consortium (N = 75,696). Bold text in final column indicates that the effect in SpiroMeta was in the same direction to the effect in UK Biobank.

**Figure 1.**
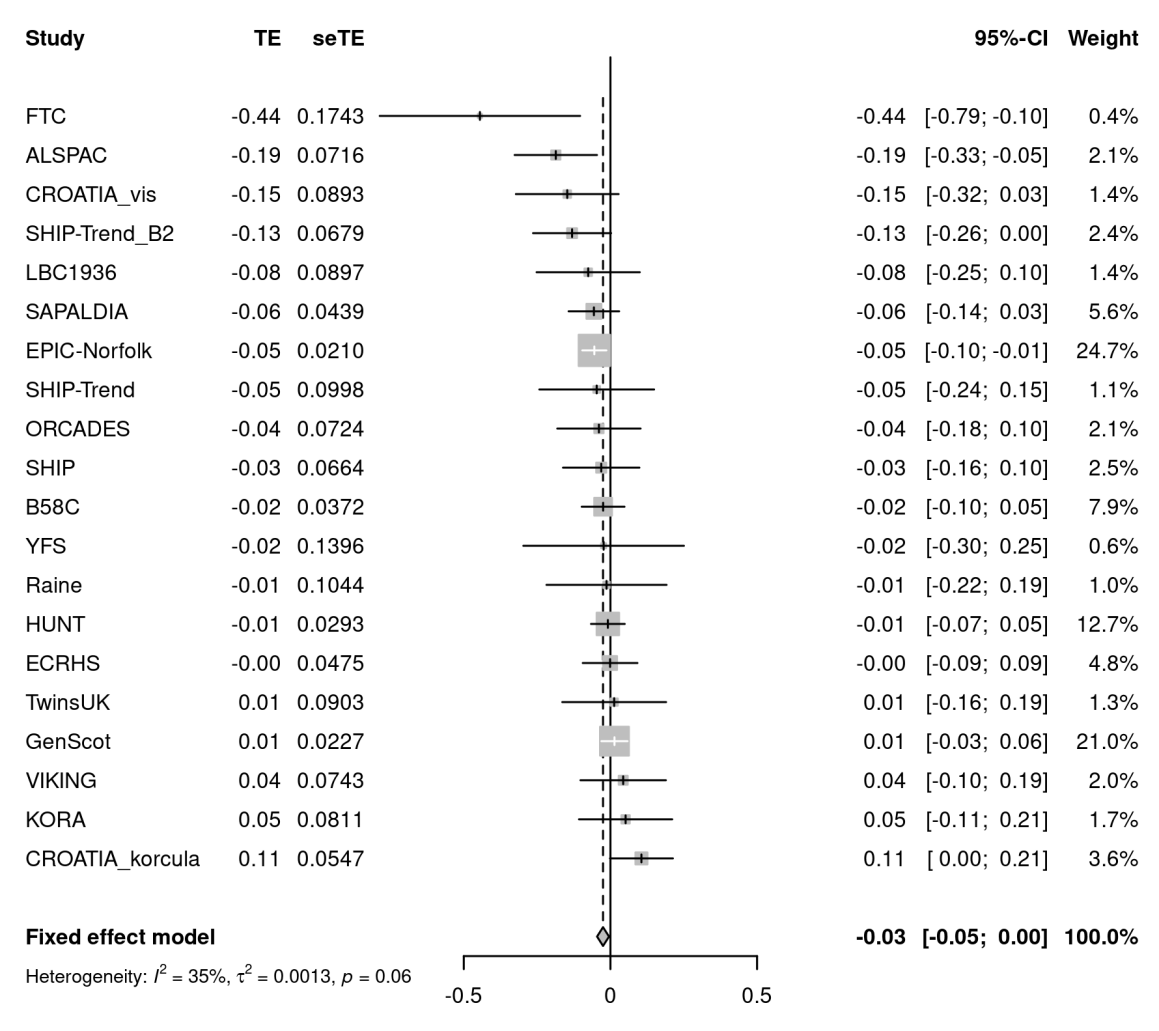
Meta-analysis of rs7697189-by-sex interaction effects on lung function in SpiroMeta cohorts. The forest plot shows the beta-coefficients (test effects, TE) and standard errors for the interaction between rs7697189 and sex on forced expiratory volume in 1 second (FEV _1_) in 20 cohorts of the SpiroMeta consortium (total N = 75,696). The overall effect size from fixed effects meta-analysis is represented by the diamond.

**Figure 2.**
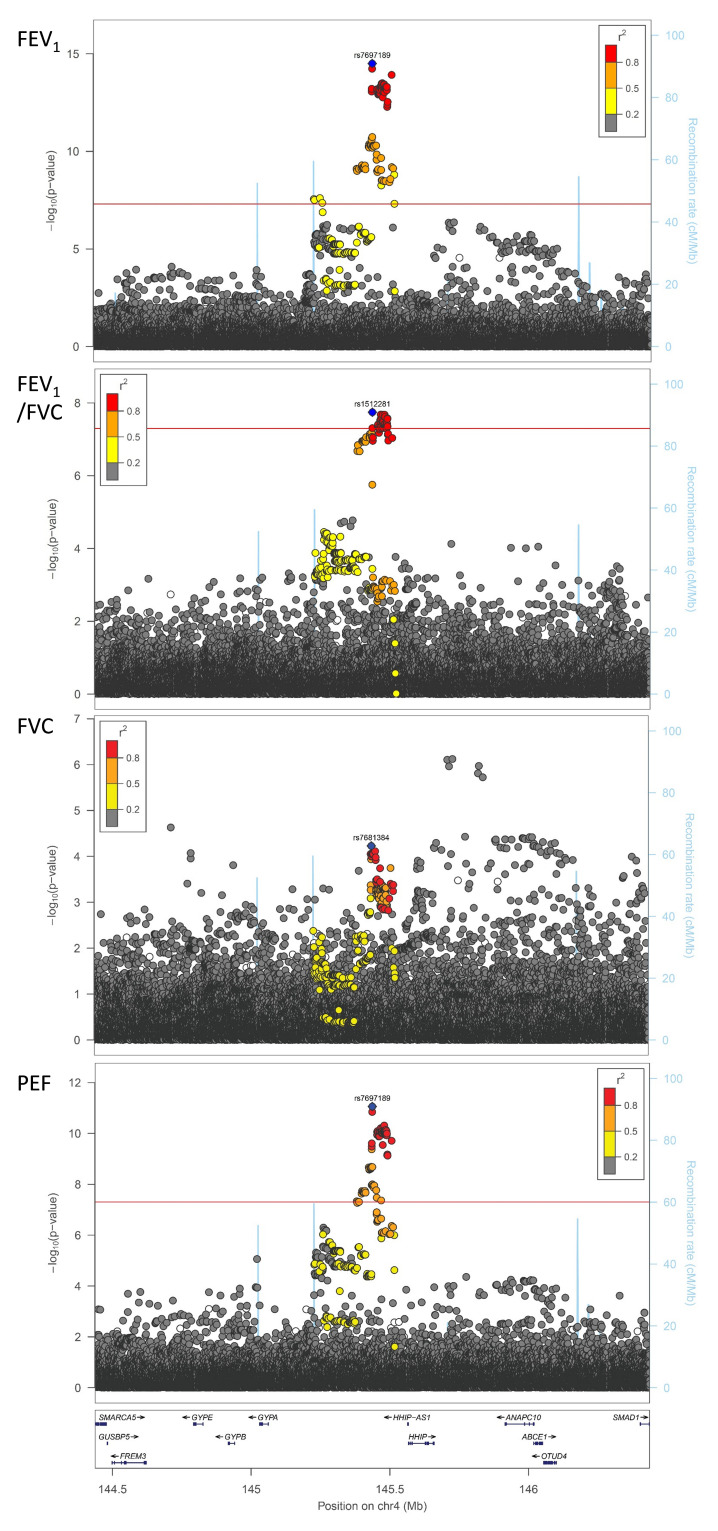
Genotype-by-sex interaction results within the *HHIP* region for lung function traits in UK Biobank. The SNP with the strongest association in the rs7697189-proximal region is represented by a blue diamond. The FEV _1_ and PEF sentinels are rs7697189, the FEV _1_/FVC sentinel is rs1512281 (R ^2^ = 0.95 with rs7697189), and the FVC sentinel is rs7681384 (R ^2^ = 0.57 with rs7697189). Note that there is an independent suggestively significant signal from rs2353939 and surrounding SNPs for FVC, but this did not replicate in SpiroMeta cohorts. All other SNVs are colour coded according to their linkage disequilibrium (R ^2^) with the sentinel SNP (as shown in the key). All imputed SNVs are plotted irrespective of MAF, demonstrating that rarer variants are not exhibiting significant interactions with sex on lung function. The locations of genes in the region are shown in the lower panel of each plot. Recombination rate is represented by the blue lines. These plots were generated using LocusZoom software.

We sought evidence for replication of all 26 signals in up to 75,696 individuals from 20 cohorts of the SpiroMeta consortium. One variant, rs76911399, was excluded because it was poorly imputed in SpiroMeta cohorts and had no directly genotyped or well-imputed proxies (at r ^2^ threshold 0.8). Of the remaining 25 signals, 19 exhibited the same direction of interaction effect as in UK Biobank. Furthermore, the effect sizes (beta coefficients) from the regression analyses of all 25 SNPs in UK Biobank and SpiroMeta showed a correlation of 0.51 (Figure S2, *Extended data* ^10^). The SNP with the strongest evidence for interaction with sex on lung function in SpiroMeta cohorts was rs7697189 (near *HHIP*) (replication interaction P = 0.016) ( Table 1, Figure 1). The minor (C) allele of rs7697189 had a larger effect on lung function in males (β = 0.052 [SE 0.004], P = 2.13 x 10 ^-33^) compared to females (β = 0.013 [SE 0.003], P = 1.16 x 10 ^-5^) ( Table 1). This SNP resides upstream of the *HHIP* gene and is in linkage disequilibrium with two previously reported lung function-associated sentinel SNPs, rs13141641 ^16, 17^ (r ^2^ = 0.91) and rs13116999 ^17^ (r ^2^ = 0.56). SNP rs7697189 also showed some evidence of interaction with sex on PEF (β = -0.035 (0.005), P = 8.78 x 10 ^-12^), FEV _1_/FVC (β = -0.028 (0.005), P = 8.98 x 10 ^-8^), and FVC (β = -0.020 (0.005), P = 8.71 x 10 ^-5^) (Table S1, *Extended data* ^10^; Figure 2).

### rs7697189 interacts with sex on lung function independently of height, smoking and pubertal timing

As SNPs in *HHIP* are also reported to be associated with height ^18^ and increased height is associated with increased lung function, it is possible that rs7697189 has differential effects on lung function in males and females through differential effects on height. However, the association of rs7697189 with standing height was not modified by sex in a combined analysis of UK Biobank males and females with a genotype-by-sex interaction term (interaction P = 0.806). We also conducted a sensitivity analysis showing that the effect of the rs7697189-by-sex interaction on FEV _1_ was consistent with the original estimate after adjustment for sitting height (β = -0.04 [SE = 0.005], P = 1.97 × 10 ^-15^).

Amongst the 303,612 UK Biobank participants in this study, the proportion of ever-smokers was higher in males (52.8%) than females (40.3%) (Table S2). A larger effect of rs7697189 on lung function in males compared to females could arise if there was an interaction effect with smoking. However, there was no interaction between rs7697189 and ever-smoking status on FEV _1_ in this study (interaction P = 0.63). Pack years data was available for 94,750 UK Biobank participants. In sensitivity analyses we found a similar rs7697189-by-sex effect size on FEV _1_ when adjusted for pack years (β = -0.033 [SE = 0.009], P = 3.50 × 10 ^-4^) and no interaction between genotype and pack years on FEV _1_ (interaction P = 0.80).

SNP rs7697189, and correlated SNPs in the region, have been shown to be associated with expression levels of *HHIP* in lung tissue ^19^. HHIP is a critical protein during early development and *HHIP* variants have been associated with lung function in infancy ^20^. We tested whether *HHIP* SNPs also have differential effects on lung function in females compared to males in childhood using data from children with an average age of eight years in the ALSPAC and Raine studies (N = 5645). In the meta-analysis of ALSPAC and Raine (Figure S3, *Extended data* ^10^), whilst we observed a point estimate for the rs7697189-by-sex interaction effect on FEV _1_ that was consistent with the confidence intervals for the discovery effect observed in UK Biobank, the confidence intervals overlapped the null (which likely reflects in part the smaller numbers studied in these cohorts). Finally, as pubertal timing has been associated with adult lung function ^21^, we tested for an effect of relative age at puberty on the association between rs7697189 and lung function in a sex-stratified analysis. The association between *HHIP* SNPs and lung function was adjusted for relative age at voice breaking in males and for age at menarche in females, but adjusted effect estimates were highly consistent with the unadjusted estimates of the SNPs on lung function (Table S3, *Extended data* ^10^).

### rs7697189 is associated with HHIP expression, but no interaction with sex

It is possible that rs7697189 interacts with sex on lung function through differential effects on *HHIP* expression. We confirmed that rs7697189 is associated with *HHIP* expression in lung tissue but we did not detect an interaction with sex on *HHIP* expression (Table S4, *Extended data* ^10^). However, *HHIP* (in all samples irrespective of genotype at rs7697189) did show differential expression between males and females, with females showing higher expression (Table S5; *Extended data* ^10^). This agrees with GTEx data on HHIP lung expression in males and females (Figure S4, *Extended data* ^10^).

### rs7697189 is in linkage disequilibrium with a SNP predicted to disrupt SREBP and SRF motifs

HaploReg v4.1 ^22^ was used to identify whether rs7697189, or SNPs in linkage disequilibrium, affected transcription factor binding motifs. This demonstrated that rs7697189 itself was predicted to change FAC1 and FOXO motifs and was within a chromatin mark indicative of enhancer activity in embryonic stem cell lines differentiated to CD56+ mesoderm and CD184+ endoderm cultured cells. A SNP (rs12504628) in complete linkage disequilibrium with rs7697189 changes SREBP and SRF motifs. These transcription factors have been reported to be involved in sex hormone signalling ^23, 24^.

## Discussion

We identified a genome-wide significant genotype-by-sex interaction signal at a locus previously reported for association with lung function upstream of the *HHIP* gene (rs7697189, FEV _1_ interaction P = 3.15 × 10 ^-15^). The SNP showed some evidence of replication in 75,696 individuals from 20 independent studies of the SpiroMeta consortium (β = -0.025 (0.01), P = 0.016), although it did not pass a Bonferroni correction for multiple testing. We demonstrated that the differential effects of this SNP in males and females (FEV _1_ β = 0.052 (0.004) in males and 0.013 (0.003) in females, corresponding to an untransformed FEV _1_ β = 0.028 [SE 0.0022] litres in males vs β = 0.009 [SE 0.0014] litres in females) did not appear to be mediated by effects on height, smoking behaviour or pubertal age.

There was evidence that SNPs at the *HHIP* locus demonstrated interactions with sex on two additional lung function traits in UK Biobank: FEV _1_/FVC and PEF (β = -0.028 (0.005), P = 8.78 × 10 ^-12^ and β = -0.035 (0.005), P = 8.78 × 10 ^-12^, respectively). Stratified analyses in males and females demonstrated that these SNPs appeared to have a stronger effect on lung function in males compared to females. There was no interaction between these SNPs and ever-smoking status on lung function in UK Biobank, suggesting that the stronger effect in males is not due to differences in smoking behaviour. We also demonstrate that an association between these SNPs and standing height is not modified by sex, suggesting that differential effects on height in males and females do not explain the genotype-by-sex interaction on lung function. It should be noted, however, that lung function is more closely related to sitting and thoracic height than standing height. We conducted sensitivity analyses showing that the rs7697189-by-sex interaction remained after adjustment for sitting height, but thoracic height was not available.

In contrast to these results, a recent study found comparatively weak evidence of an interaction effect between a SNP (rs13140176) in high LD with rs7697189 (r ^2^ = 0.93) and sex on risk of COPD in UK Biobank ^25^. This is likely in part to be due to reduced power to detect interaction effects on a binary trait. Indeed, in our study, the rs13140176-by-sex interaction effect on FEV _1_/FVC passes the conventional threshold for genome-wide significance (P<5×10 ^-8^) but when COPD was defined as FEV _1_/FVC<0.7 this threshold was not met (P=0.023). Nevertheless, rs13140176 shows a consistent direction of effect between the studies: the lung function-lowering allele increases risk of COPD to a greater extent in males than females ^25^.

The genome-wide significant sex interaction locus is located upstream of the *HHIP* gene, a region previously reported to be associated with lung function ^12, 15^ and *HHIP* gene expression ^19^. The *HHIP* gene encodes hedgehog-interacting protein, a negative regulator of hedgehog signalling. The hedgehog signalling pathway regulates numerous physiological processes such as growth, self-renewal, cell survival, differentiation, migration, and tissue polarity and plays a vital role in the morphogenesis of lung and other organs ^26^. Hedgehog signalling has also been shown to participate in regulation of stem and progenitor cell populations in adult tissues, impacting tissue homeostasis and repair ^27^. SNP rs7697189, showing the strongest sex interaction on lung function in our study, is in strong linkage disequilibrium (R ^2^ = 0.93) with SNPs residing in an *HHIP* enhancer region ^19^. These enhancer-region SNPs were reported to be associated with enhancer activity and *HHIP* expression in lung tissues. They also exhibit genome-wide significant genotype-by-sex interactions on lung function in our data. We therefore tested the effect of rs7697189 on *HHIP* expression in lung tissue from 472 males and 566 females to look for sex differential effects. In contrast to the previous study ^19^, we found that the lung-function lowering G allele was associated with enhanced expression of *HHIP* in both males and females, and that expression was lower in males than females. However, the association between rs7697189 and *HHIP* expression was not modified by sex. This may be because there is no sex differential effect on expression, or the study might have been underpowered to detect an interaction effect. It is also possible that sex-differential effects of *HHIP* SNPs are only detectable in particular cell types. We therefore propose that *HHIP* eQTLs could be tested in larger numbers of males and females and in different cell types. Our *in silico* analyses predict that rs7697189 and a SNP in linkage disequilibrium (rs12504628) change transcription factor motifs that may be relevant to the effect of sex hormones on lung development, but experimental analyses will be required to test these hypotheses.

Investigating the effects of HHIP at different stages of development by sex may help to shed light on its mechanism of action. In our study we had access to genetic and lung function data from 5645 children with an average age of eight years. Though underpowered to detect the association between rs7697189 and FEV _1_ seen in UK Biobank adults, the lack of a similar trend in children suggests that *HHIP* variants may have differential effects at different developmental stages (though the genotype-by-sex interaction is in the same direction as in adults). We also looked for an effect of timing of puberty on the association between rs7697189 ** and lung function in adults, but adjustment for relative age of voice breaking in males and relative age at menarche in females made no difference to the relationship between rs7697189 and lung function. As UK Biobank participants were aged between 40 and 69 years at recruitment, we did not have the longitudinal data to investigate the effect of *HHIP* SNPs on trajectories of lung function decline throughout life ^28^, but this could be an interesting area for future studies.

We identified four additional genome-wide significant (interaction P<5x10 ^-8^) sex-by-genotype interactions on lung function in our discovery analysis in UK Biobank, with a further 21 that met a less stringent threshold of interaction (P<1x10 ^-6^). As far as we are aware, this is the first genome-wide sex-by-genotype interaction study for lung function traits. We did not find a significant genotype-by-sex interaction on lung function or COPD at the *CELSR1* locus (interaction P = 0.525 and P = 0.503, respectively) previously reported to have sex-specific effects on risk of COPD ^8^.

In conclusion, we have identified a novel genotype-by-sex interaction at SNPs at a putative enhancer region upstream of the hedgehog-interacting protein ( *HHIP*) gene. Establishing the mechanism by which *HHIP* has sex differential effects on lung function will be important for our understanding of the biological underpinnings of COPD in males and females. This knowledge, in turn, will be crucial to optimising treatment in males and females.

## Materials and Methods

### Ethics and consent

This study used anonymised data from UK Biobank (RRID: SCR_012815), which comprises over 500,000 volunteer participants aged 40–69 years recruited across Great Britain between 2006 and 2010. The protocol and consent were approved by the UK Biobank’s Research Ethics Committee. Our analysis was conducted under approved UK Biobank data application number 648. For SpiroMeta consortium cohorts, all participants provided written informed consent and studies were approved by local Research Ethics Committees and/or Institutional Review boards. Full ethics statements for each SpiroMeta consortium cohort is included in the S1 Appendix ( *Extended data*, ^10^).

### UK Biobank

The UK Biobank is described here: http://www.ukbiobank.ac.uk. Individuals were included in this study if (i) they had no missing data for sex, age, height, and smoking status, (ii) their spirometry data passed quality control, as described previously ^7^, (iii) their genetically inferred sex matched their reported sex, (iv) they had genome-wide imputed genetic data, (v) they were of genetically determined European ancestry, and (vi) they were not first- or second-degree relatives of any other individual included in the study. In total, 303,612 individuals met these criteria (Table S2, *Extended data* ^10^).

Participants’ DNA was genotyped using either the Affymetrix Axiom ^®^ UK BiLEVE array or the Affymetrix Axiom ^®^ UK Biobank array ^29^. Genotypes were imputed based on the Human Reference Consortium (HRC) panel, as described elsewhere ^29^. Variants with minor allele frequency (MAF)<0.01 were excluded, as were variants with imputation quality scores <0.3.

### SpiroMeta consortium

The SpiroMeta consortium meta-analysis comprised 75,696 individuals from 20 studies (see S1 Appendix for details, *Extended data* ^10^). Ten studies (N=17,280) were imputed using 1000 Genomes Phase 1 reference panel ^30, 31^, nine (N=37,919) were imputed using the Haplotype Reference Consortium (HRC) panel ^29^, and one (N=2077) was imputed using the HapMap CEU Build 36 Release 22. The ALSPAC (RRID: SCR_007260) and Raine studies also provided data on children with an average age of eight years (N=4426 and N=1219, respectively). Tables S6 and S7 show definitions of all abbreviations, study characteristics, details of genotyping platforms and imputation panels and methods ( *Extended data* ^10^). Measurements of spirometry for each study are as previously described ^7, 21^. Fourteen SpiroMeta studies had data on PEF (N=51,555).

### Statistical analysis

Spirometry-based lung function traits FEV _1_, FEV _1_/FVC, FVC, and PEF were pre-adjusted for age, age ^2^, standing height (or sitting height in the sensitivity analysis) and smoking status and the residuals rank-transformed to normality using the rntransform function of the GenABEL package (RRID: SCR_001842) in R (RRID: SCR_001905). To test each imputed autosomal variant for an interaction effect, a linear regression model with genotype (additive effect), sex, genotype-by-sex interaction, genotyping array and the first ten principal components included as covariates was implemented using Plink 2.0 software (RRID: SCR_001757). Step-wise conditional analyses to identify independently associated variants were undertaken using GCTA software ^32, 33^.

Regression analysis to test genotype-by-sex interactions on height were conducted using a model including genotype (additive effect), age, age ^2^, sex, genotyping array and the first ten principal components as covariates. Interactions between smoking status and genotype on lung function were tested using lung function traits transformed as described above (with sex included in the model instead of ever-smoking status). The linear regression model included genotype (additive effect), ever-smoking status, a genotype-by-smoking interaction term, genotyping array and the first ten principal components.

To test whether pubertal timing has differential effects on the association between SNPs and lung function in males and females, the regression model was adjusted for relative age at menarche in females and relative age at voice breaking in males. Relative age at voice breaking is categorised as earlier than average (1), around average (2) and later than average (3) in UK Biobank. Age at menarche is given as the participant’s age at menarche in years. To make these variables comparable, age at menarche was categorised as early (<12 years old), average (12–14 years old) and late (>14 years old) as in a previous study ^34^. As in the lung function analyses, ancestry-based principal components and genotyping array were included in all the regression models.

For the SpiroMeta consortium, summary statistics were generated by each contributing cohort separately according to the same analysis plan as the UK Biobank data. Meta-analysis of SpiroMeta cohorts was conducted using inverse-variance weighted fixed effects meta-analysis using the metagen function of the meta package in R.

### The lung eQTL study

The lung expression quantitative trait loci (eQTL) study database has been described previously ^35– 37^ and in S1 Appendix ( *Extended data* ^10^). *HHIP* differential gene expression analysis between females and males was performed using linear regression. Association of rs7697189 and rs7697189-by-sex interaction with gene expression was tested in 1,038 subjects with genotypes using MatrixEQTL package in R. All analyses were done separately in Laval, UBC and Groningen, and then combined using a meta-analysis with fixed-effects model and inverse-variance weights.

## Data availability

### Underlying data

UK Biobank data is an open access resource available to bona fide researchers undertaking health-related research. Researchers must apply for access (see https://www.ukbiobank.ac.uk/researchers/ for more details). Genome-wide interaction study summary statistics are available on Figshare (see below).

Figshare: Genome-wide sex interaction study summary statistics for lung function traits in UK Biobank. https://doi.org/10.6084/m9.figshare.12298736.v1 ^38^

### Extended data

Figshare: Variants associated with *HHIP* expression have sex-differential effects on lung function: supplementary material. https://doi.org/10.6084/m9.figshare.12129207 ^10^

This project contains Fawcett_et_al_Extended_data_supplement.docx, which contains the following extended data:

- Supplementary materials and methods
- Figure S1. Genome-wide interaction SNP-by-sex interaction results on four measures of lung function in UK Biobank
- Figure S2. Correlation between genotype-by-sex interaction effect sizes in UK Biobank and the SpiroMeta studies
- Figure S3. Association between rs7697189 and FEV _1_ in children from the ALSPAC and Raine cohorts
- Figure S4. GTEx data on expression of HHIP by sex in different tissues
- Table S1. Association between rs7697189 and lung function traits in males and females, and genotype-by-sex interaction results
- Table S2. UK Biobank demographics
- Table S3. Sex-stratified association between rs7697189 and lung function before and after adjustment for pubertal timing
- Table S4. Association between rs7697189 and *HHIP* expression and rs7697189-by-sex interaction on *HHIP* expression
- Table S5. Differential expression of *HHIP* in males compared to females
- Table S6. SpiroMeta studies
- Table S7. SpiroMeta analysis methods

Data are available under the terms of the Creative Commons Zero "No rights reserved" data waiver (CC0 1.0 Public domain dedication).
