# Supplementary material for: Variants associated with HHIP expression have sex-differential effects on lung function
Source: Wellcome Open Res. 2021 May 24;5:111. Originally published 2020 Jun 1. [Version 2] doi: 10.12688/wellcomeopenres.15846.2 (PMC7938335; doi:10.12688/wellcomeopenres.15846.2)
Supplement: Peer review history

# Peer review: Reviewer response for version 1

**DOI:** 10.21956/wellcomeopenres.17382.r42679

**Published:** 2021-03-05

**Authors:** Eistine Boateng

**Affiliations:** 1 Early Life Origins of Chronic Lung Diseases, Research Center Borstel, Leibniz Lung Center, Member of the German Center for Lung Research (DZL), Borstel, Germany

In this study, the authors attempted to explain genotype-by-sex interaction on lung function. The membrane protein, hedgehog interacting protein (HHIP), is reported as a susceptibility factor for COPD. Thus, SNPs upstream regulate the expression of HHIP, which is evidently decreased in COPD tissues as shown in related studies. The manuscript is well written and findings could serve as a fundamental basis for future experimental studies. However, I have a few comments which could be considered to improve the impact of the study.

**Comments**: One key finding in this study was increased expression of (HHIP) in lung tissues from females compared to those from males. Authors should further explain or speculate possible reasons for this observation.

 The level of HHIP is known to be decreased in lung tissues from COPD patients. How could the results of this interaction study translate to the molecular pathogenesis of lung diseases eg. COPD *vis*-à- *vis* its prevalence in males and females at the study sites where samples were obtained? Again, the development of COPD is characterized by different stages. What is the relevance of the study to staging of lung diseases between males and females?

 Lung function partly reflects on the biological state of the organ and authors appear to propose that HHIP may exhibit sex differential effects on lung function. Could authors add a brief outlook for future experimental studies which may want to follow up on their findings? For example, are there differences in cell-specific expression of HHIP in the lungs of males and females and how can this relate to the pathogenesis and risk of lung diseases between the sexes?

Is the work clearly and accurately presented and does it cite the current literature?

Yes

If applicable, is the statistical analysis and its interpretation appropriate?

I cannot comment. A qualified statistician is required.

Are all the source data underlying the results available to ensure full reproducibility?

Yes

Is the study design appropriate and is the work technically sound?

Yes

Are the conclusions drawn adequately supported by the results?

Yes

Are sufficient details of methods and analysis provided to allow replication by others?

Yes

Reviewer Expertise:

Pulmonary fibrosis, COPD, asthma, lung development, and miRNA

I confirm that I have read this submission and believe that I have an appropriate level of expertise to confirm that it is of an acceptable scientific standard.

# Response

**Published:** 2021-05-18

**Authors:** Katherine Fawcett

**Affiliations:** University of Leicester, UK

We are very grateful to the reviewer for taking the time to read and comment on our manuscript.  We will address the comments on a point-by-point basis:

*One key finding in this study was increased expression of (HHIP) in lung tissues from females compared to those from males. Authors should further explain or speculate possible reasons for this observation.*

 The higher expression of *HHIP *in females is intriguing, but it is not clear (from our reading of the literature) why this might be.

*The level of HHIP is known to be decreased in lung tissues from COPD patients. How could the results of this interaction study translate to the molecular pathogenesis of lung diseases eg. COPD vis-à-vis its prevalence in males and females at the study sites where samples were obtained? Again, the development of COPD is characterized by different stages. What is the relevance of the study to staging of lung diseases between males and females?*

 In our study, the lung-function-lowering allele of rs7697189 was associated with increased expression of *HHIP*.  This is the opposite direction of effect to that found in the Zhou et al. paper cited in the discussion, but is consistent with other reports (van der Plaat DA, de Jong K, Lahousse L, Faiz A, Vonk JM, van Diemen CC, Nedeljkovic I, Amin N, Brusselle GG, Hofman A, Brandsma CA, Bossé Y, Sin DD, Nickle DC, van Duijn CM, Postma DS, Boezen HM. Genome-wide association study on the FEV1/FVC ratio in never-smokers identifies HHIP and FAM13A. J Allergy Clin Immunol. 2017 Feb;139(2):533-540. doi: 10.1016/j.jaci.2016.06.062. Epub 2016 Sep 6. PMID: 27612410; Morrow, Jarrett D et al. “Functional interactors of three genome-wide association study genes are differentially expressed in severe chronic obstructive pulmonary disease lung tissue.” Scientific reports vol. 7 44232. 13 Mar. 2017, doi:10.1038/srep44232; Lamontagne, Maxime et al. “Refining susceptibility loci of chronic obstructive pulmonary disease with lung eqtls.” PloS one vol. 8,7 e70220. 30 Jul. 2013, doi:10.1371/journal.pone.0070220) that show COPD risk alleles associated with increased *HHIP *expression .  It is possible that, given males have lower expression of *HHIP *than females, studies of COPD with a higher proportion of males amongst cases than controls could find spurious associations between lower *HHIP *expression and COPD.

 We assume that by "staging of lung diseases" the reviewer is referring to the different trajectories of COPD as outlined, for example, by Lange et al. (Lange P, Celli B, Agustí A, Boje Jensen G, Divo M, Faner R, Guerra S, Marott JL, Martinez FD, Martinez-Camblor P, Meek P, Owen CA, Petersen H, Pinto-Plata V, Schnohr P, Sood A, Soriano JB, Tesfaigzi Y, Vestbo J. Lung-Function Trajectories Leading to Chronic Obstructive Pulmonary Disease. N Engl J Med. 2015 Jul 9;373(2):111-22. doi: 10.1056/NEJMoa1411532. PMID: 26154786).  Without understanding the mechanism by which HHIP affects lung function, it is not clear how it would impact trajectories of lung disease.  In our discussion, we suggest longitudinal studies to test the effects of *HHIP *SNPs on trajectories of lung function decline in males and females.

 "As UK Biobank participants were aged between 40 and 69 years at recruitment, we did not have the longitudinal data to investigate the effect of *HHIP *SNPs on trajectories of lung function decline throughout life (28) , but this could be an interesting area for future studies."

*Lung function partly reflects on the biological state of the organ and authors appear to propose that HHIP may exhibit sex differential effects on lung function. Could authors add a brief outlook for future experimental studies which may want to follow up on their findings? For example, are there differences in cell-specific expression of HHIP in the lungs of males and females and how can this relate to the pathogenesis and risk of lung diseases between the sexes?*

 We do suggest a number of possible avenues for follow up.  Though we find no interaction between *HHIP *SNPs and sex on *HHIP *expression, our sample size for the expression analysis was quite small.  Testing for interaction in larger datasets would therefore be warranted.  We also suggest investigating sex differential effects of *HHIP *at different developmental stages and on trajectories of lung function in longitudinal studies.  Finally, we propose testing our in silico-generated hypothesis that "rs7697189 and a SNP in linkage disequilibrium (rs12504628) change transcription factor motifs that may be relevant to the effect of sex hormones on lung development".  We are not aware of any cell-type specific datasets that would allow comparison of *HHIP *expression between males and females, but if these were available then they could indeed throw light on the mechanism of action of sex-differential effects of HHIP.  We have added the following to the discusssion:

 "It is also possible that sex-differential effects of *HHIP *SNPs are only detectable in particular cell types.  We therefore propose that *HHIP *eQTLs could be tested in larger numbers of males and females and in different cell types."

# Peer review: Reviewer response for version 1

**DOI:** 10.21956/wellcomeopenres.17382.r39582

**Published:** 2020-07-21

**Authors:** David M. Mannino

**Affiliations:** 1 Department of Preventive Medicine and Environmental Health, University of Kentucky College of Public Health, Lexington, KY, USA

The authors provide an interesting analysis of the gene/sex interaction affect on lung function as demonstrated in the UK Biobank cohort and validated in the Spirometa consortium. They found a greater affect in males than females ( 28 mL vs 9 mL).

 Comments: The authors note that this gene is also related to height. Although they adjusted for standing height (and height squared) in the analysis they should note in the limitations that lung function is actually more closely related to sitting height (or even better- thoracic height) which is not well measured. Thus - it is possible that the difference could be explained by other factors related to how we estimate "normal" lung function.

Is the work clearly and accurately presented and does it cite the current literature?

Yes

If applicable, is the statistical analysis and its interpretation appropriate?

Yes

Are all the source data underlying the results available to ensure full reproducibility?

Yes

Is the study design appropriate and is the work technically sound?

Yes

Are the conclusions drawn adequately supported by the results?

Yes

Are sufficient details of methods and analysis provided to allow replication by others?

Yes

Reviewer Expertise:

Pulmonary function, COPD

I confirm that I have read this submission and believe that I have an appropriate level of expertise to confirm that it is of an acceptable scientific standard.

# Response

**Published:** 2021-05-18

**Authors:** Katherine Fawcett

**Affiliations:** University of Leicester, UK

We would like to thank the reviewer for taking the time to review our manuscript.  We agree that sitting and thoracic height are more related to lung function than standing height and have therefore added the following to the discussion:

 "It should be noted, however, that lung function is more closely related to sitting and thoracic height than standing height.  We conducted sensitivity analyses showing that the rs7697189-by-sex interaction remained after adjustment for sitting height, but thoracic height was not available."
